# Decision Making about Risk of Infection by Young Adults with CF

**DOI:** 10.1155/2013/658638

**Published:** 2013-01-10

**Authors:** Lisa Reynolds, Gary Latchford, Alistair J. A. Duff, Miles Denton, Tim Lee, Daniel Peckham

**Affiliations:** ^1^Leeds Institute of Health Sciences, University of Leeds, Leeds LS2 9LJ, UK; ^2^Adult Cystic Fibrosis Unit, Leeds Teaching Hospitals NHS Trust, Leeds LS9 7TF, UK; ^3^Department of Clinical and Health Psychology, Leeds Teaching Hospitals NHS Trust, Leeds LS9 7TF, UK; ^4^Paediatric Cystic Fibrosis Centre, Leeds Teaching Hospitals NHS Trust, Leeds LS1 3EX, UK; ^5^Microbiology, Leeds Teaching Hospitals NHS Trust, Leeds LS9 7TF, UK

## Abstract

Young people with cystic fibrosis (CF) are asked to avoid a number of environments associated with increased infection risk, but in practice they need to balance this with competing priorities such as building and sustaining relationships with friends and family. This study explored the process by which young people make these decisions. Mixed methods were used: a vignette study presenting choices around engaging in activities involving a degree of infection risk and a thematic analysis of participant's accounts of their decision making. The eight participants chose to engage in high risk behaviours in 59% of the choices. All participants chose to engage in at least one risky behavior, though this was less likely when the risk was significant. Thematic analysis revealed large areas of misunderstanding and lack of knowledge, leading to some potentially worrying misconceptions about the nature of infections and risk. Young people with CF are not currently making informed decisions around activities that involve increased risk of infection, and there is an urgent need for CF teams to address this in information provision.

## 1. Introduction

Cystic fibrosis (CF) is the most common transmitted genetic disease affecting Caucasians. It is characterised by a defect in the cystic fibrosis transmembrane conductance regulator (CFTR) gene which controls chloride transport in cells. This leads to a buildup of thick and sticky mucous, causing numerous complications, particularly in the respiratory and digestive systems. It also significantly increases susceptibility to respiratory infection, with subsequent lung damage and eventual mortality.

CF is life limiting and previously considered a paediatric condition, but advances in treatment have witnessed a gradual increase in life expectancy [1] and a corresponding focus on the impact of the condition on psychosocial functioning in young adulthood. The treatment burden in CF is extremely intrusive, and adherence to CF medication in young people is a topic of understandable interest [2]. Less well studied is adherence to guidelines on reducing risk of infection.

Significant risks are posed by infections such as *Burkholderia cepacia* complex (*Bcc*) and *Pseudomonas aeruginosa* (*PsA*) [3, 4], some strains of which are highly contagious, and for people with CF known to cause exacerbations in respiratory symptoms and increased morbidity. Efforts to reduce infection rates include complete segregation of all patients with CF in clinic to reduce cross infection. Particular infections are associated with particular environments. Known environmental sources of *PsA*, the most prevalent source of infection for people with CF, include plants, soil, environments contaminated by human or animal waste, surface water, and spa baths or Jacuzzis [5].

Young people with cystic fibrosis (CF) are expected to consider infection risk when making decisions about careers, leisure, and social activities. And current infection control guidelines recommend that people with CF avoid environments associated with increased risk of contracting them [5–8]. Adherence to these guidelines depends upon a good understanding of the nature of the risk, as well as motivation to follow them in the context of the competing demands of young adulthood, such as forming relationships. As yet, the process of decision making in this area remains unexplored.

This study examined the decision making of a small group of young people with CF concerning exposure to risk of infection, and the beliefs and knowledge guiding their choices.

## 2. Methods

### 2.1. Participants

Eight young people with CF, aged 16–25 years, were recruited through out-patient appointments at Leeds Regional Adult CF unit, Leeds, UK. This age group was selected as young people are considered to become more increasingly autonomous in their decision making over 15 years of age [9]. All participants had a FEV1 above 30% at the time of the study. *PsA* infection status was recorded.

Ethical approval for this study was granted by the Leeds East Research Ethics Committee on 09/09/2009.

### 2.2. Measures

Seven vignettes were written with a consultant microbiologist, with reference to current guidelines on infection control in CF [5–8]. Each vignette contained at least one decision about exposure to an environment associated with increased risk of infection, but in the context of a potentially desirable activity. For example, one described a situation in which a patient is asked by someone they find attractive if they would like to join them in going horse riding, which could increase risk of exposure to *Aspergillus*. A subsequent choice (in this example, to visit stables with them) could increase this risk still further. The vignettes are summarised in Table 1. 

Vignettes have been widely used in studies exploring medical decision making [10] and this methodology has been found to have a high level of internal validity and consistency amongst participants in a number of studies [11].

### 2.3. Procedure

Participants consenting to take part in the study were interviewed via telephone, at a mutually convenient prearranged time. They were read each vignette and asked to make the choices contained within them, and to describe their reasoning as they did so using a “think aloud” technique [12]. After the vignettes were presented, participants were asked for their views on the extent to which risks associated with infection should be considered in their career and lifestyle choices. Finally the participants were asked about their knowledge of *PsA* and *Bcc* infections. The length of the interviews varied between 20 and 45 minutes.

All conversations were recorded, transcribed, and subjected to thematic analysis [13]. Thematic analysis is a well-used method for the identification of themes in qualitative data without attachment to any specific theoretical framework. This allowed factors influencing decision making to be identified for each participant, as well as themes common across participants.

## 3. Results

### 3.1. Participants

Table 2 shows details of participant demographics, including *PsA* infection status, FEV_1_% predicted and BMI (based on an average from measurements taken in the previous year), and number of episodes of IV antibiotics in the previous year. None of the participants had a history of *Burkholderia cepacia* complex infection.

### 3.2. Vignettes

The decisions made by each participant on the vignettes are shown below in Table 3.

The vignettes contained four choices associated with average risk, and ten with increased risk, including six judged as significant by a consultant microbiologist (MD). The eight participants choose to engage in the behaviour associated with increased risk 47/80 times (59%). On 28 occasions a participant chose not to engage with the behaviour citing risk as the reason. Three decisions not to engage in a risky behaviour were made because the participant said they were not interested in the activity rather than that a concern about possible risk. Two participants were unable to reach a decision for one vignette (5b, concerning meeting someone with CF who had *PsA*).

Examining the pattern of responses, two of the choices (involving increased risk of exposure to *PsA* and MRSA) were made by all participants. One participant made all of the increased risk choices. All participants chose to engage in the behaviour associated with increased risk on at least one occasion, but tended to be less likely to do so when this was significant, indicating some discrimination between level of risk.

Four of the eight participants had chronic *PsA*, and their responses to the vignettes featuring a significant increase of risk of *PsA* (to self or others) were compared to those with intermittent or no *PsA*. The chronic *PsA* participants chose to engage in the increased risk behaviour on fewer occasions than did the intermittent or absent group (8 and 13 resp.).

### 3.3. Thematic Analysis

The analysis revealed seven main themes within the participants' descriptions of how they made the decisions: Knowledge, Previous experience, Medical influence, Social influence, Living a life, Achieving a balance, and Decision-making strategies. Six of these were common to all eight participants; one (“Achieving a balance”) to seven, indicating a broad consensus in how the participants viewed the factors influencing their choices. The themes are described in more detail below.

**Theme 1 (Knowledge)**

This theme emerged as participants described their understanding of CF and infection risk when explaining their decisions. In general, even when there was a general awareness of risk, participants were unaware of the precise nature of this, or the likely impact of an infection on them. Some participants held particular misconceptions about infection, such as the necessity for exposure to risky situations in order to build resistance:

*“Whereas if you go out every now and again and you get mixing with people with bugs … they're not really bad and you do not really catch it, then you're going to sort of build your immune system a little bit, aren't you?” (participant 1)*

In addition, a number of participants were confused about the nature of different infections, with a number unable to describe *Bcc* and one participant describing it as a more severe case of *PsA*. There was, though, an awareness that having CF resulted in vulnerability to infections known to be harmful and a need to protect oneself.

**Theme 2 (Previous experience)**

This theme is central in understanding how participants might have acquired information about their CF and risk of infections. It includes comments about segregation which all had experienced as adults, though none were able to articulate a detailed understanding of the reasons why segregation had been enforced. A number of participants also commented on their previous or current experience of infection, referring to this when making decisions. This risk assessment was not always accurate, with some underestimating the impact that a previous infection had had on their health:

*“I grow MRSA like in my cough sputum … other people can catch it from hospitals and things and it can be really harmful, but as far as I know … I think I've grown it for years and it's never, never really affected my health.” (participant 4)*

Where there was no direct experience of an infection participants appeared to have limited knowledge and understanding of where it might be found or the impact it may have.

**Theme 3 (Medical influences)**

When participants were aware of advice provided by healthcare professionals on infection, risk they considered this in reaching their decision:

*“I know that the hospital do not quite like us all mixing up, because obviously one bug is more dangerous to one person than another.” (participant 1)*

When they had a clear understanding, their decision tended to be based on level of risk. However even when there was some awareness of a healthcare message, participants' understanding of the reasons for this advice was sometimes limited.

**Theme 4 (Social influences)**

Social factors, including the influence of friends and family, appeared to have a significant influence on participants' decision making. Where infection risk was not apparently considered, social factors were often cited as a reason for the decision.

*“You go on holiday to have fun with your friends, so it's all about having fun.” (participant 5)*

Moreover, social factors were at times considered more important than infection risk in choosing to act. In some of the vignettes this was a conscious altruistic act—for example, to visit a sick friend or family member in hospital despite the personal risk. In other vignettes the desire to have fun with friends seemed the most salient factor. The supportive role of family was often mentioned, as was the tendency of parents to give advice. There was some reference to the emergence of greater independence with age and that this sometimes led to tension with family members around decisions.

**Theme 5 (Living a life)**

The importance of having a life that includes pleasant or “normal” experiences was often cited as an influence on decisions:

*“it's all about not missing out.” (participant 7)*

The potential personal benefits of making the choices were often cited before infection risk or health was considered. Although there were individual differences in the way in which personal reasons influenced choices, in general they centred on personal interest and enjoyment, and a desire not to be excluded socially from activities with friends or family. Some participants also discussed their plans for the future and a desire for a career or family.

**Theme 6 (Achieving a balance)**

This theme emerged as participants attempted to evaluate and minimise risk in decision making, whilst still participating in activities they find enjoyable. It is characterised by attempts to assess risk, though this often proved difficult because of limited understanding or limited information on which to base a decision, and some participants clearly found avoiding all risk impracticable:

*“at the end of the day you cannot sort of shut yourself away … I think it's really important to think about infection, but I think some people could take it too far.” (participant 1)*

Risk to health was often actively weighed against the potential benefits of engaging in the activity and the desired lifestyle this represented. This balancing process was often moderated by input from others, particularly family members. When describing this process, some participants reflected on the nature of this balance, of having a fulfilled life relative to having a healthy life.

**Theme 7 (Decision-making strategies)**

Participants employed several strategies to help them arrive at a decision and feel comfortable with their choice. Sometimes this was simply to focus exclusively on the enjoyable aspects of the situation and not dwell on the decision at all. Another strategy was to engage in the activity despite awareness of the potential risk but attempt to minimise this, for example, by washing hands in the hospital vignette or meeting another CF patient in an open environment rather than enclosed space. Finally, when struggling to make a choice some participants said they would leave the final decision with the others mentioned in the vignette and follow their advice:

*“I would tell them what I thought, but leave it up to their decision as to whether we met up or not.” (participant 6)*

The presence of such strategies to reduce discomfort with the choice reinforces the idea that there was often an awareness of some level of risk, even when this was not well understood.

## 4. Discussion

This study has revealed something of the dilemmas young people with CF face when making decisions about lifestyle and balancing infection risk with the desire to lead normal, fulfilling lives. All participants chose at least one option involving increased risk in the vignettes and many chose several, though this was generally less likely in the vignettes associated with a significantly increased risk. Participants often explained their choices by referring to the anticipated pleasures of taking part. When this was with a group of friends they were particularly fearful of social exclusion because of their illness. In the hospital vignettes they also talked of their sense of obligation to put family and friends above any personal danger, making it clear that there was a rationale behind their choice, although lack of knowledge suggests it was unlikely to be a well-informed one.

Of particular concern were the many instances where participants showed that they did not understand the nature or severity of risk in the vignettes. This lack of knowledge had sometimes led to our young people holding particularly dangerous misconceptions around CF and infection risk, such as the belief that exposure to infection increase resistance expressed by participant one. This included misconceptions about the seriousness of their own experiences of infection. Psychological models of decision making around health behaviour such as the Health Belief model [14] stress the importance of the perceived susceptibility and seriousness of the health threat, together with the perceived benefit of taking action. If the threat is underestimated and the advantages of action minimised, as is true in the responses to many of the vignettes in this study, then action is unlikely.

The use of defensive strategies—minimising risk, placing external responsibility for the decision on others and avoidance of thinking—suggests that there is enough of an awareness of risk to produce discomfort. Analysis also revealed that advice from others—the CF team and parents—often played a part in decision making, though this was not straightforward. The Theory of Planned Behavior [15] highlights the potentially important role played by subjective norms (perceived social pressure) in decision making, and the general willingness to consider medical advice reported by many of the participants suggests that they may be amenable to an intervention to increase knowledge. The main message from the study, however, is that these young people are not currently making informed decisions.

The current study had a relatively small sample size, but the use of mixed methods does allow for a more in-depth analysis of the process of decision making as well as an overview of the degree of risk-taking indicated by these participants. The participants were all young adults and it is not known whether similar decisions would be made by different age groups, or whether the processes influencing choice would be similar. Young adults were thought to be a suitable target group because they have recently developed autonomy for decision making and are typically facing many social demands around friendship groups and developing relationships. In addition, it was thought that the results of this study might be able to inform information giving in the transition process.

The main clinical implication of this study is that teams need to address gaps in knowledge about infection risk with young people. This should form part of preparation for transition. Transition represents an opportunity to engage young people in discussions about risk and to correct any misconceptions that they hold.

There are clear indications from this research that young people may be receptive to this, in that there is already a general awareness. There is no evidence that people with CF are generally prone to risk taking around health [16]. Improved knowledge will not necessarily lead to better (or safer) decision making, of course, and the current study highlights the complex nature of these decisions and the competing demands involved. It seems good practice, therefore, that real life scenarios are used when discussing the practical implications of infection risk, and that the information is conveyed in a style which invites honest questioning. Creative solutions may be needed in finding ways that to address the very real concerns that these young people have about social isolation and maintaining relationships with family and peers. Ultimately young people may still opt to prioritise social contact over caution, but this will at least be an informed decision.

## Figures and Tables

**Table 1:** Description of vignettes and source and level of risk.

Vignette	Situation	Source of risk	Level of risk
1a	Attend a family wedding	Cross-infection	Average
1b	Discover there are over 100 guests attending	Cross-infection	Average
2a	Go swimming with friends	*PsA *	Slightly increased
2b	Use a Jacuzzi with friends	*PsA *	Significantly increased
3a	Go camping with friends	*PsA, Aspergillus *	Average
3b	Use a communal shower/toilet	*PsA, Aspergillus *	Average
4a	Go horse riding	*Aspergillus *	Increased
4b	Visit stables	*Aspergillus *	Significantly increased
5a	Meet someone with CF after talking on facebook	*PsA, Bcc *	Significantly increased
5b	Be in close-proximity to someone with CF who has *Pseudomonas *	*PsA, Bcc *	Significantly increased
5c	Share a taxi with someone with CF	*PsA, Bcc *	Significantly increased
6a	Visit a friend in hospital	MRSA	Increased
6b	Visit a friend who has MRSA	MRSA	Significantly increased
7	Visit a relative in a nursing home	MRSA	Increased

*PsA*: *Pseudomonas  aeruginosa*; *Bcc*: *Burkholderia cepacia* complex; MRSA: Methicillin-resistant *Staphylococcus aureus*.

**Table 2:** Participant demographics.

No.	Age	Gender	FEV_1_% predicted	BMI	IV antibiotics	*Pseudomonas* status
1	21	Female	50.70	21.67	2	Intermittent *PsA *
2	18	Male	78.66	22.29	0	Intermittent *PsA *
3	24	Female	97.20	20.12	0	Intermittent *PsA *
4	18	Female	63.00	21.75	0	Non-*PsA *
5	17	Female	68.11	23.13	4	Chronic *PsA *
6	23	Female	36.64	17.57	3	Chronic *PsA *
7	21	Female	33.86	21.88	7	Chronic *PsA *
8	24	Male	59.20	21.27	1	Chronic *PsA *

FEV_1_% predicted and BMI are averages for the previous year; IV antibiotics are number of episodes in previous year.

**Table 3:** Participant responses to vignettes.

Vignette	Risk as judged by consultant microbiologist	Agree to participate in activity	Avoid—because of risk	Avoid—other reason	Unsure
1a	Average	8	0	0	0
1b	Average	8	0	0	0
2a	Slightly increased	8	0	0	0
2b	Significantly increased	5	2	1	0
3a	Average	6	2	0	0
3b	Average	6	2	0	0
4a	Increased	6	1	1	0
4b	Significantly increased	4	2	1	0
5a	Significantly increased	2	3	0	2
5b	Significantly increased	3	5	0	0
5c	Significantly increased	3	5	0	0
6a	Increased	6	2	0	0
6b	Significantly increased	2	6	0	0
7	Increased	8	0	0	0

## References

[1] Dodge JA, Lewis PA, Stanton M, Wilsher J (2007). Cystic fibrosis mortality and survival in the UK: 1947–2003. *European Respiratory Journal*.

[2] Bernard RS, Cohen LL (2004). Increasing adherence to cystic fibrosis treatment: a systematic review of behavioural techniques. *Pediatric Pulmonology*.

[3] Frederiksen B, Lanng S, Koch C, Hoiby N (1996). Improved survival in the Danish centre-treated cystic fibrosis patient: results of aggressive treatment. *Pediatric Pulmonology*.

[4] Pamukcu A, Bush A, Buchdahl R (1995). Effects of *Pseudomonas aeruginosa* colonization on lung function and anthropometric variables in children with cystic fibrosis. *Pediatric Pulmonology*.

[5] Cystic Fibrosis Trust (2004). *Pseudomonas Aeruginosa Infection in People with Cystic Fibrosis, Suggestions for Prevention and Infection Control*.

[6] Saiman L, Siegel J (2003). Infection control recommendations for patients with cystic fibrosis: microbiology, important pathogens, and infection control practices to prevent patient-to-patient transmission. *Infection Control and Hospital Epidemiology*.

[7] Cystic Fibrosis Trust (2004). *The Burkholderia Cepacia Complex, Suggestions for Prevention and Infection Control*.

[8] Cystic Fibrosis Trust

[9] Wray-Lake L, Crouter AC, McHale SM (2010). Developmental patterns in decision-making autonomy across middle childhood and adolescence: European American parents’ perspectives. *Child Development*.

[10] Bachmann LM, Mühleisen A, Bock A, ter Riet G, Held U, Kessels AGH (2008). Vignette studies of medical choice and judgement to study caregivers’ medical decision behaviour: systematic review. *BMC Medical Research Methodology*.

[11] Ryan M (2004). Discrete choice experiments in health care. NICE should consider using them for patient centred evaluations of technologies. Editorial. *British Medical Journal*.

[12] Gilhooly K, Green C, Richardson JTE (1996). Protocol analysis: theoretical background. *Handbook of Qualitative Research Methods for Psychology and the Social Sciences*.

[13] Braun V, Clarke V (2006). Using thematic analysis in psychology. *Qualitative Research in Psychology*.

[14] Becker MH (1974). *The Health Belief Model and Personal Health Behaviour*.

[15] Ajzen I (1991). The theory of planned behavior. *Organizational Behavior and Human Decision Processes*.

[16] McEwan FA, Hodson ME, Simmonds NJ (2011). The prevalence of “risky behaviour” in adults with cystic fibrosis. *Journal of Cystic Fibrosis*.

